# Supplementary material for: A cognitive representation in primary visual cortex modulated by vision
Source: bioRxiv. 2026 Sep 3:2026.08.29.748029. Preprint. [Version 1] doi: 10.64898/2026.08.29.748029 (PMC13684629; doi:10.64898/2026.08.29.748029)
Supplement: Supplementary file 1 [file NIHPP2026.08.29.748029v1-supplement-1.pdf]

bioRxiv preprint doi: https://doi.org/10.64898/2026.08.29.748029; this version posted September 3, 2026. The copyright holder for this preprint (which was not certified by peer review) is the author/funder, who has granted bioRxiv a license to display the preprint in perpetuity. It is made available under a CC-BY 4.0 International license.

Supplementary figures

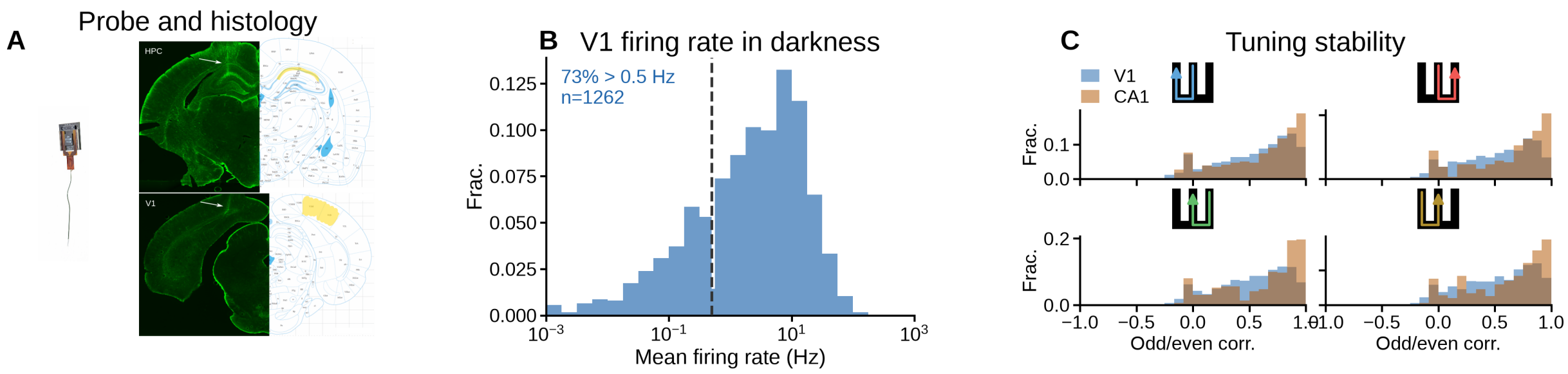

fig. S1. (A) Left: Flexible polyimide probe used in this study. Right: histology showing the implantation locations in dorsal hippocampus CA1 (top) and V1 (bottom). White arrows in GFAP staining (green) mark the probe tracks. **(B)** Distribution of mean firing rate of V1 neurons in darkness during the W-maze spatial alternation task. Only movement periods (>4 cm/s) are included. Dashed line marks 0.5 Hz, used as threshold for defining dark-active vs -inactive groups. **(C)** Distribution of stability of spatial tuning (defined as the correlation between tuning curves learned from even and odd trials) in darkness in each of the four path types, for both V1 (blue) and CA1 (orange).

bioRxiv preprint doi: https://doi.org/10.64898/2026.08.29.748029; this version posted September 3, 2026. The copyright holder for this preprint (which was not certified by peer review) is the author/funder, who has granted bioRxiv a license to display the preprint in perpetuity. It is made available under a CC-BY 4.0 International license.

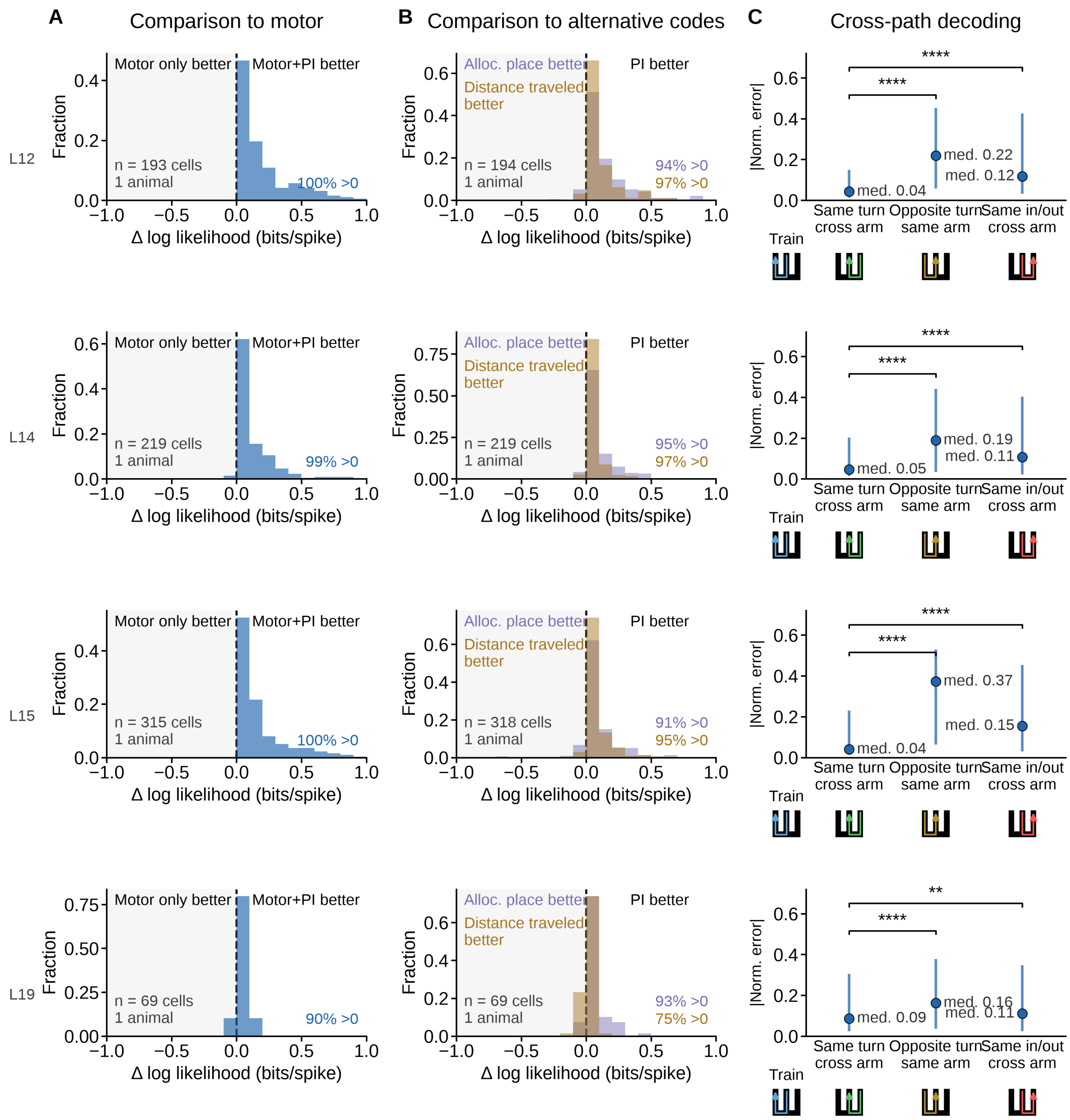

fig. S2. (A) Per-animal data of Fig. 1E. **(B)** Per-animal data of Fig. 1F. **(C)** Per-animal data of Fig. 1G.

bioRxiv preprint doi: https://doi.org/10.64898/2026.08.29.748029; this version posted September 3, 2026. The copyright holder for this preprint (which was not certified by peer review) is the author/funder, who has granted bioRxiv a license to display the preprint in perpetuity. It is made available under a CC-BY 4.0 International license.

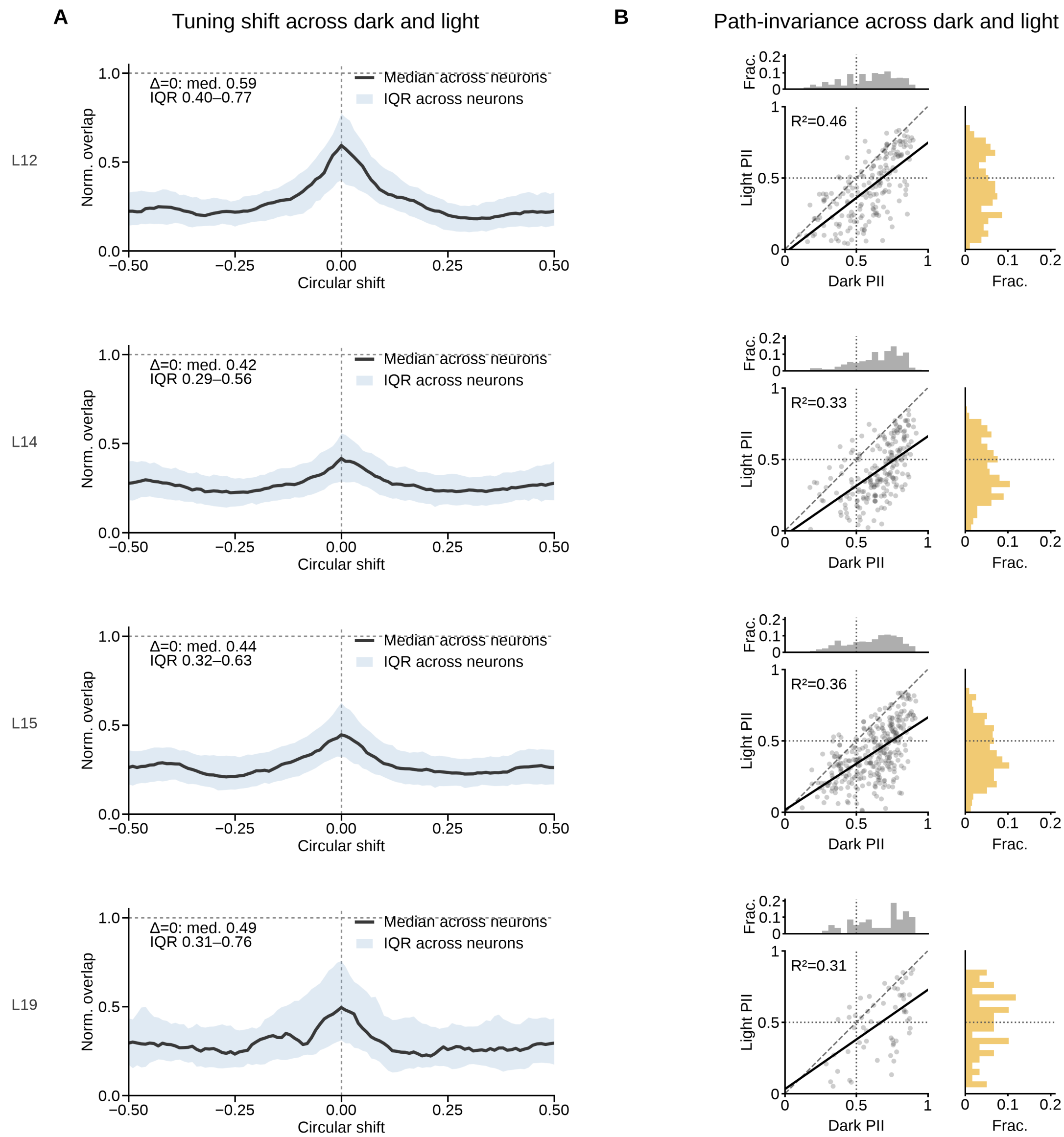

fig. S3. (A) Per-animal data of Fig. 2B. **(B)** Per-animal data of Fig. 2C.

bioRxiv preprint doi: https://doi.org/10.64898/2026.08.29.748029; this version posted September 3, 2026. The copyright holder for this preprint (which was not certified by peer review) is the author/funder, who has granted bioRxiv a license to display the preprint in perpetuity. It is made available under a CC-BY 4.0 International license.

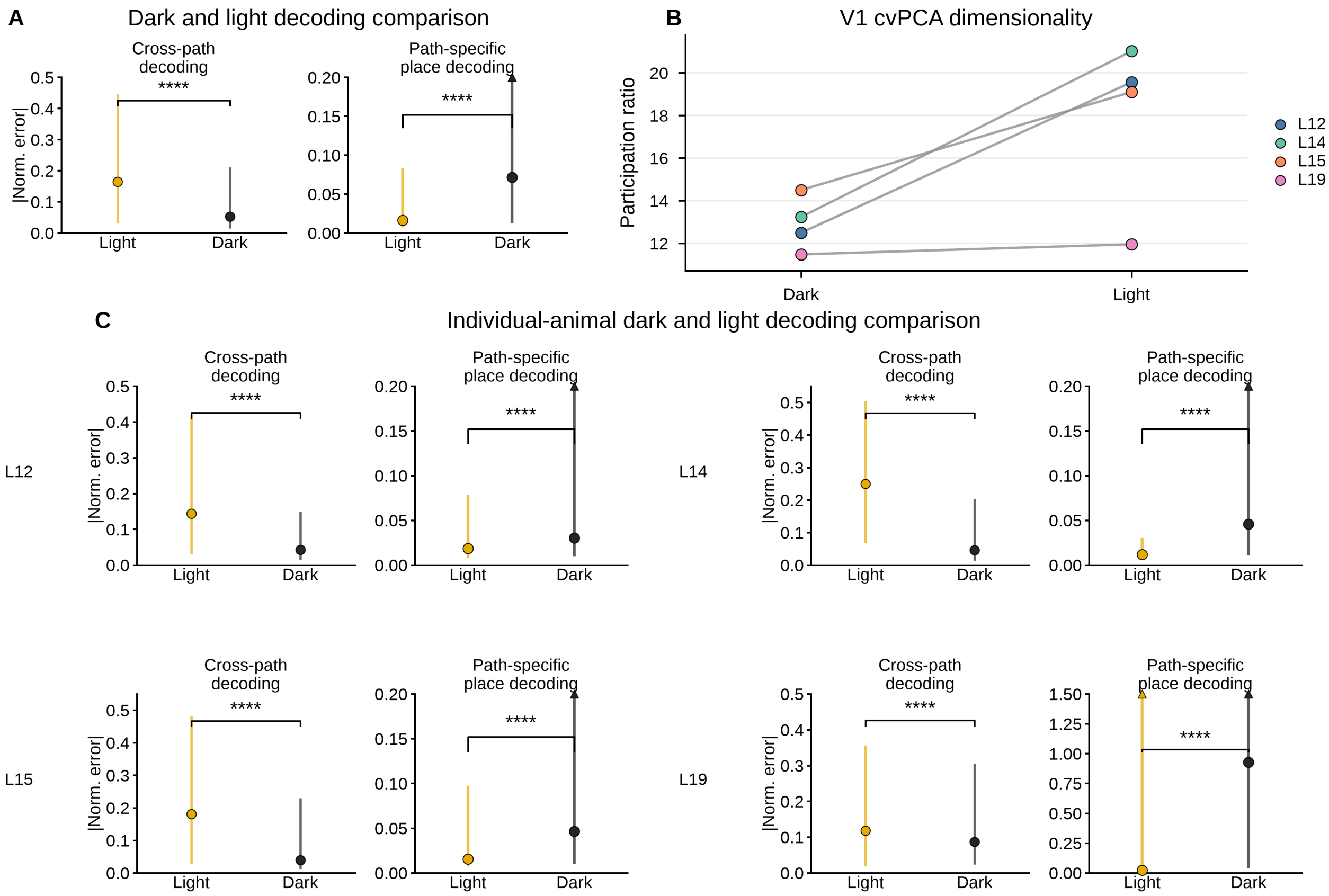

fig. S4. (A) Cross-path decoding of same-turn paths (left) and path-specific place decoding (right) in light and dark. Compared with light, cross-path decoding error is lower in darkness, whereas path-specific place decoding error is higher, consistent with light-induced reduction in path-invariance. **(B)** Paired comparison of V1 population dimensionality between dark and light epochs, quantified as the participation ratio of cross-validated PCA spectra computed from tuning curves across the four paths. Colors indicate different animals. **(C)** Per-animal data for panel A. In panel A, significance denotes the largest per-animal *p*-value when the effect direction was consistent across animals; panel C shows animal-specific tests. *$p<0.05$, **$p<10^{-2}$, ***$p<10^{-3}$, ****$p<10^{-4}$.

bioRxiv preprint doi: https://doi.org/10.64898/2026.08.29.748029; this version posted September 3, 2026. The copyright holder for this preprint (which was not certified by peer review) is the author/funder, who has granted bioRxiv a license to display the preprint in perpetuity. It is made available under a CC-BY 4.0 International license.

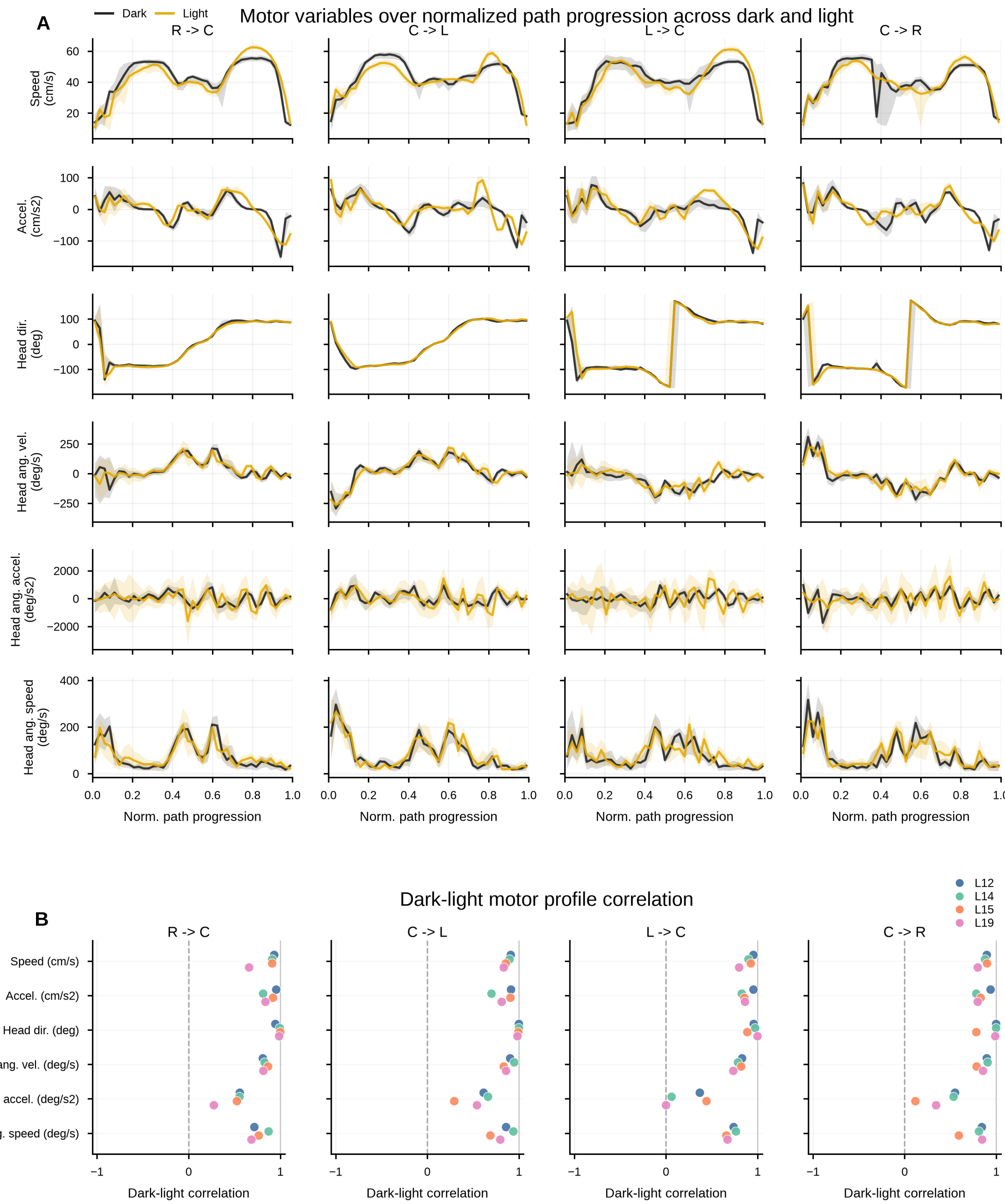

fig. S5. **(A)** Six motor variables aligned to normalized path progression for each of the four paths in light (yellow) and dark (black) epochs for a single animal (L14). Solid lines show medians and shaded bands show interquartile ranges across trials. **(B)** Correlation of the motor variables between light and dark epochs. Colors indicate different animals.

bioRxiv preprint doi: https://doi.org/10.64898/2026.08.29.748029; this version posted September 3, 2026. The copyright holder for this preprint (which was not certified by peer review) is the author/funder, who has granted bioRxiv a license to display the preprint in perpetuity. It is made available under a CC-BY 4.0 International license.

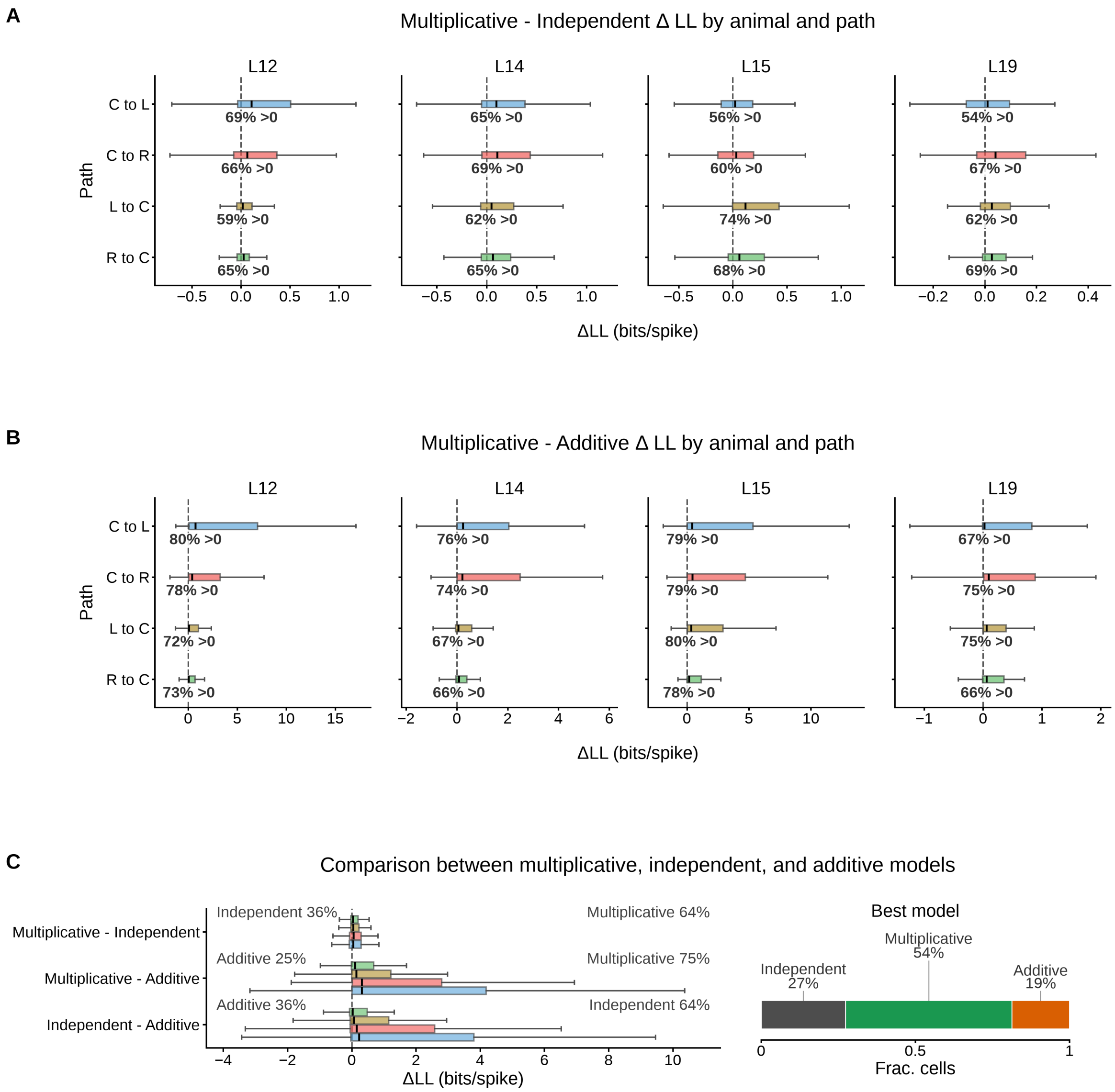

fig. S6. (A) Difference in log likelihood between the multiplicative and independent models (defined in Fig. 3A) for all four path types for each of the four animals used in this study (L12, L14, L15, and L19). Colors indicate different path types. The number to the right of each box-and-whisker plot indicates the fraction of cells with ΔLL greater than 0 (favoring multiplicative model). **(B)** Same as (A) but for multiplicative and additive models. **(C)** Left: Three-way comparison of pair-wise differences in log likelihood among multiplicative, independent, and additive models. Right: Fraction of V1 neurons best explained by each of the three models.

bioRxiv preprint doi: https://doi.org/10.64898/2026.08.29.748029; this version posted September 3, 2026. The copyright holder for this preprint (which was not certified by peer review) is the author/funder, who has granted bioRxiv a license to display the preprint in perpetuity. It is made available under a CC-BY 4.0 International license.

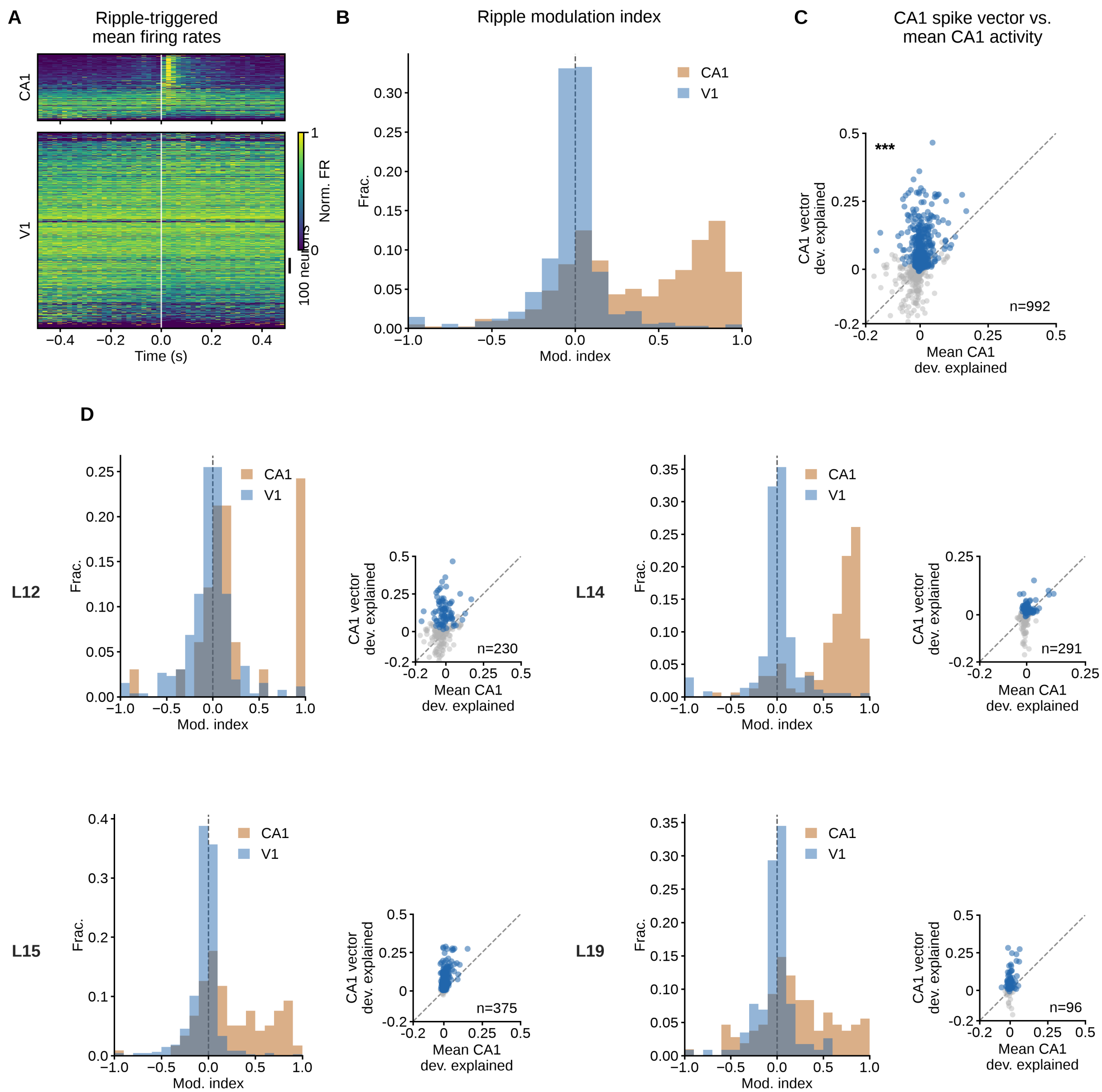

fig. S7. (**A**) Heatmap of mean ripple-triggered firing-rate across awake ripples. The modulation is very strong for CA1 neurons but much weaker for V1 neurons. **(B)** Distributions of ripple modulation indices for V1 (blue) and CA1 (orange) neurons during light epochs, pooled across sessions. For each neuron, the index was calculated as ((R-B)/(R+B)), where R and B are the mean peri-ripple firing rates from 0 to 100 ms and −500 to −300 ms relative to ripple onset, respectively. (**C**) Comparison of GLM prediction when using CA1 spike vectors vs. mean CA1 activity. Deviance explained for mean CA1 activity is mostly at 0 whereas CA1 vector-based prediction is much higher. The dashed line indicates equal performance; blue points denote units significantly predicted by the population-vector model in the shuffle test (p<0.05), and gray points denote nonsignificant units. Significance was assessed using a one-sided, 100,000-permutation linear mixed-effects test of the paired model difference with animal as a random effect. ***$p<10^{-3}$. **(D)** Per-animal data of panels B and C.

bioRxiv preprint doi: https://doi.org/10.64898/2026.08.29.748029; this version posted September 3, 2026. The copyright holder for this preprint (which was not certified by peer review) is the author/funder, who has granted bioRxiv a license to display the preprint in perpetuity. It is made available under a CC-BY 4.0 International license.

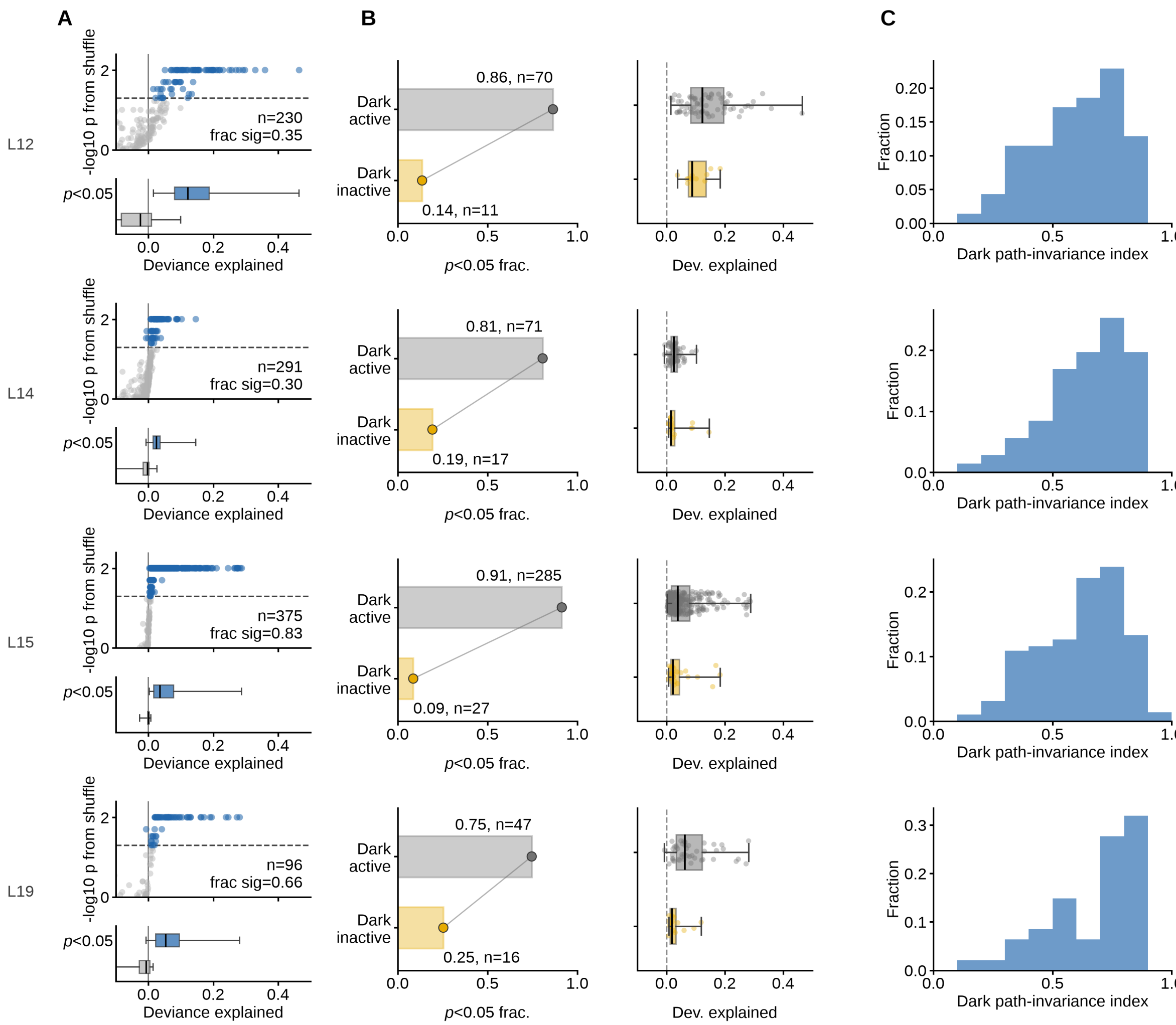

fig. S8. (**A**) Per-animal data of Fig. 4D. **(B)** Per-animal data of Fig. 4E. **(C)** Per-animal data of Fig. 4F.
